# Locoregional Therapies for Hepatocellular Carcinoma in Patients with Nonalcoholic Fatty Liver Disease

**DOI:** 10.3390/biomedicines12102226

**Published:** 2024-09-30

**Authors:** Stephen Susman, Breanna Santoso, Mina S. Makary

**Affiliations:** 1 Department of Radiology, Yale University Medical Center, New Haven, CT 06510, USA; 2 Heritage College of Osteopathic Medicine, Ohio University, Dublin, OH 43016, USA; 3 Department of Radiology, The Ohio State University Wexner Medical Center, Columbus, OH 43202, USA

**Keywords:** hepatocellular carcinoma, nonalcoholic fatty liver disease, nonalcoholic steatohepatitis, metabolic syndrome, transarterial embolization, chemoembolization, radioembolization, TACE, TAE, TARE

## Abstract

Hepatocellular carcinoma (HCC) is the third most common cause of cancer-related death worldwide with an average five-year survival rate in the US of 19.6%. With the advent of HBV and HCV treatment and prevention, along with the rising rates of obesity, nonalcoholic fatty liver disease (NAFLD) and metabolic syndrome are set to overtake infectious causes as the most common cause of HCC. While surgical resection and transplantation can be curative when amenable, the disease is most commonly unresectable on presentation, and other treatment approaches are the mainstay of therapy. In these patients, locoregional therapies have evolved as a vital tool in both palliation for advanced disease and as a bridge to surgical resection and transplantation. In this review, we will be exploring the primary locoregional therapies for HCC in patients with NAFLD, including transarterial chemoembolization (TACE), bland transarterial embolization (TAE), transarterial radioembolization (TARE), and percutaneous ablation.

## 1. Introduction

### 1.1. NAFLD

Hepatocellular carcinoma (HCC) is the most common cause of primary liver cancer, making up 90–95% of hepatic malignancies [1]. It is the third most common [2] and most rapidly increasing cause of cancer-related malignancy worldwide [3], contributing to 9.1% of all cancer-related deaths [1]. The 5-year survival rate in select patients who receive the first-line treatment approaches a 70% 5-year survival, in optimal candidates [4]. Unfortunately, the vast majority of HCC is caught at an advanced stage in patients who are not ideal candidates for surgery, with a resulting less than 20% expected 5-year survival [2,5].

The most common risk factor is cirrhosis, which arises from infection, chronic toxic exposure, genetic conditions, and metabolic diseases [1]. In the West, Hepatitis C (HCV), alcohol-associated liver disease, and nonalcoholic fatty liver disease (NAFLD) are the most common risk factors. While the individual risk of HCC secondary to NAFLD (NAFLD-HCC) alone is low, at 0.08–0.63 per 1000 person-years, NAFLD can progress to nonalcoholic steatohepatitis (NASH). In this group, as many as 25% then go on to develop NAFLD-related cirrhosis [6]. Of those who develop cirrhosis, 10–15% then go on to develop HCC [7]. Additionally, unlike many other causes of HCC, between 14 and 37% of NAFLD-HCC occurs outside of the context of cirrhosis [8,9]. Most concerningly, the prevalence of NAFLD risk factors is growing rapidly in the West, and it has become the most common cause of liver disfunction worldwide [10], increasing from 15% of the US population in 2005 to 25% in 2015 [8]. As NAFLD incidence continues to increase, NAFLD is expected to overcome HCV in the US as the most common cause of HCC in many countries in the world by 2030 [7,11].

Screening in NAFLD patients unfortunately has unique challenges, as both elements of a robust screening program are limited. Specifically, as NAFLD patients often develop HCC without first developing symptoms of cirrhosis, it is infeasible to screen the estimated 80 million people with radiographic evidence of NAFLD in the US [12]. Additionally, as the sensitivity of ultrasonography is notoriously operator-dependent, examination in patients with obesity often is limited. As a result, HCC often is diagnosed either incidentally or at a more advanced stage [13].

### 1.2. NAFLD-HCC Treatment Overview

Surgical resection is the first-line treatment for BCLC 0 or A patients with solitary tumors, no extrahepatic metastasis or large vessel involvement, and normal liver function. While those who qualify for this potentially curative procedure have a 5-year survival rate of 41–74% [14], less than 10% of patients fulfilled the preoperative criteria [15]. In very early and early NAFLD-HCC (BCLC 0 and A), percutaneous ablation is also of curative intent with similar outcomes to resection [16]. This can be seen on Table 1 in the first and second columns. For unresectable HCC, orthotopic liver transplantation (OLT) is the other surgical option. To be eligible for OLT, the patient must meet the Milan criteria by having (1) a single tumor of less than 5 cm or up to three tumors each with a diameter of less than 3 cm, (2) no extrahepatic metastasis, and (3) no major vessel involvement [17]. Under these conditions, the outcomes for OLT demonstrate a remarkable 70–90% 5-year survival rate [17,18], which is similar to the expected survival rates of those undergoing OLT without HCC [19].

Unfortunately, most HCC cases are diagnosed at later stages, in patients who are not surgical candidates. NAFLD-HCC especially often presents later in life. Additionally, NAFLD is associated with comorbidities including obesity, diabetes, cardiovascular disease, metabolic syndrome, cardiovascular disease, and obstructive sleep apnea, each of which may limit certain therapeutic options available [21,22,23,24]. For instance, metabolic syndrome more than doubles the perioperative risk of death [25]. While NAFLD-HCC tends to have superior outcomes for those who qualify for surgical resection [15,26], they are likely to present at later stages, with less than 10% meeting the criteria for resection [15]. In addition, multiple cohort studies have indicated that NASH makes HCC more resistant to immunotherapy, possibly secondary to NASH-related T-cell activation, which is theorized to limit the immune response [27,28]. This all results in patients with NAFLD-HCC being associated with a shorter survival time, more advanced tumor stage, and lower possibility of receiving OLT compared with other causes [21].

For patients with NAFLD-HCC who do not qualify for surgical resection, locoregional therapies (LRTs) play a vital role in treatment. Additionally, certain modalities such as percutaneous ablation are frequently curative and have similar outcomes as surgical resection, with the additional benefit of avoiding the high perioperative morbidity and mortality associated with surgical approaches [16]. LRTs are also useful for downstaging or “bridging” patients who do not qualify for liver transplant to then become eligible [29]. Other indications include inducing contralateral healthy liver parenchymal hypertrophy before resection and palliation [2]. This review will give an overview of the major LRTs offered by vascular interventional radiologists used in the treatment of NAFLD-HCC, including the mechanism, indications, contraindications, complications, and outcomes for percutaneous ablation, transarterial embolization (TAE), transarterial chemoembolization (TACE), and transarterial radioembolization (TARE).

## 2. Percutaneous Ablation

**Figure 1:**
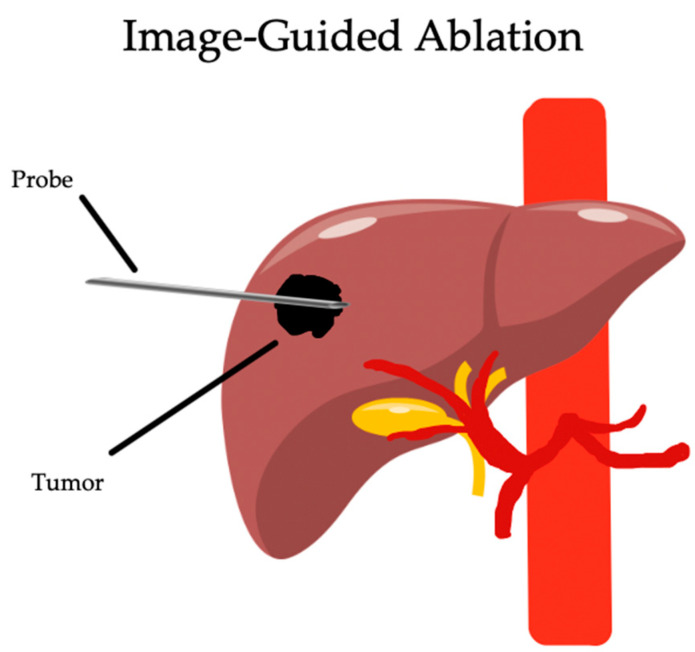
Localized destruction utilizing radiofrequency energy, microwave energy, or cryoablation.

While surgical resection or OLT is the mainstay for HCC with BCLC stage 0 and A, the majority of patients are not amenable to surgical intervention secondary to comorbidities, portal hypertension, poor hepatic function, cardiovascular risk, or the inability to tolerate prolonged general anesthesia (GA) [15]. This is especially useful for NAFLD-HCC, as their comorbidities frequently limit surgical eligibility. In this group, ablation lies as the foundation of curative intent therapy. As visualized on Figure 1, needle access to the tumor is obtained guided by imaging, with localized tissue destruction. The most common approaches function through thermal injury, including radiofrequency ablation (RFA), microwave ablation (MWA), and cryoablation (CA). Other nonthermal approaches include inducing pulses of high voltage through irreversible electroporation (IE) or through mechanical destruction with high-intensity focused ultrasound (HIFU) and histotripsy [30]. A summary of the indications, contraindications, and outcomes of percutaneous ablation for NAFLD-HCC can be seen on Table 2. The advantages and disadvantages of each LRT can be compared directly on Table 3. The choice of imaging guidance, including ultrasound (US), computed tomography (CT), and magnetic resonance imaging (MRI), is determined by individual patient characteristics and institutional availability. This review will discuss the three most common approaches: RFA, WMA, and CA.

### 2.1. Radiofrequency Ablation

#### 2.1.1. Overview

The most well-studied ablation technique is RFA, in which the probe produces an electrical current in the tissues, which causes heat generation through the Joule Effect [31]. This results in the highly localized destruction of tissues, with a goal of burning the tumor plus 5–10 mm of surrounding liver parenchyma. In time, this causes a fibrotic retraction of the necrotic tissue, which is visible radiographically as a homogenous, non-enhancing, well-circumscribed area [32]. Unfortunately, the electrical current flow of RFA is impeded near 100 C, which can limit the size of thermal ablation [33]. Additionally, RFA is relatively ineffective near vessels greater than 3 mm. This does, however, result in reducing the energy delivery to important structures, including bile ducts and large vessels [34]. This is called the heat sink effect.

**Table 2:** Summary for LRTs for NAFLD-HCC.

Modality	Indications	Contraindications	Treatment Outcomes in HCC	Treatment Outcomes in NAFLD-HCC
Percutaneous ablation	NAFLD-HCC with BCLC stage 0 and A, nonsurgical candidates [31]	Vascular invasion, intrahepatic biliary tree dilation, exophytic tumor location, uncorrectable coagulopathy, tumor is surgically resectable [2]	1- and 3-year OS was 95.8% and 71.4% 1- and 3-year DFS was 85.9% and 64.1% [35] mRECIST-CR 47% mRECIST-PR 39% [36]	Similar [37]
TAE	HCC with BCLC stage B and C; less acute toxicity [38]	Decompensated cirrhosis, reduced portal vein flow, PVT creatinine clearance < 30 mL/min, high tumor burden, untreated esophageal varices, elevated LFT marker [39]	1- and 3-year OS was 84.8% and 38.3% [40] Median PFS was 7.2 months [41] mRECIST-CR was 18.4% mRECIST-PR was 28.8% [40]	No direct studies
TACE	Intermediate, unresectable NAFLD-HCC; downstaging for OLT [42]	Decompensated cirrhosis, reduced portal vein flow, creatinine clearance < 30 mL/min, bi-lobar tumor involvement [3]	1- and 3-year OS was 89.9% and, 66.3% [43] Median PFS was 13.5 months [44] mRECIST-CR was 47.3% mRECIST-PR was 67.4% [41]	Similar [45]
TARE	Intermediate, unresectable NAFLD-HCC; no limitations on PVT; downstaging for OLT [46,47]	Decompensated cirrhosis, creatinine clearance < 30 mL/min, bi-lobar tumor involvement [48]	1- and 3-year OS was 63% and 27% [49] Median PFS was 14.5 months [50] mRECIST-CR was 13.7% mRECIST-PR was 43.1% [51]	Similar [52] Similar [53]

#### 2.1.2. Indications and Contraindications

RFA is currently indicated for NAFLD-HCC smaller than 20 mm. To qualify for RFA with curative intent, the patient must have a single nodule < 5 cm in size or up to three nodules < 3 cm in size, with no portal vein thrombus or metastasis [30]. Multiple studies have assessed RFA’s efficacy compared with surgical resection, demonstrating similar overall outcomes [30,54]. For tumors 3–5 cm in diameter in patients with HCC staged BCLC B, there is good evidence to combine ablation with TACE; however, this is not considered curative [46,55,56]. While RFA used to be the most commonly used ablative therapy, MWA has since overtaken this approach due to its unique characteristics.

Absolute contraindications include having a surgically resectable tumor and/or transplantation status, vascular invasion of the tumor, having a location less than 1 cm from the main biliary duct, intrahepatic biliary tree dilation, exophytic tumor location, and uncorrectable coagulopathy. Relative contraindications include extrahepatic metastasis, bilioenteric anastomosis, superficial/subcapsular lesions, Child–Pugh Score (CPS) C or decompensated liver disease, tumors difficult to reach, a single tumor greater than 5 cm in diameter, multiple lesions > 3 cm in diameter, more than three tumors, or tumors adjacent to hepatic vasculature [2]. Additionally, ablation is contraindicated in patients who are unable to tolerate general anesthesia. This creates a unique limitation on NAFLD-HCC patients, as NAFLD is associated with a higher rate of comorbidities that preclude general anesthesia, including severe OSA and OHS [23]. Complications include skin burns, incomplete ablation due to the heat sink effect, and postablation syndrome, a self-limiting condition, defined as nausea, vomiting, abdominal pain, and elevated liver enzymes secondary to the localized necrosis [57].

#### 2.1.3. Outcomes

For tumors < 2 cm (BCLC 0), RFA had similar overall survival outcomes to hepatic resection [16], with one study demonstrating a cure rate of between 20 and 30%, which is similar to that of resection [22]. In one systematic review, five retrospective studies totaling 639 cases were studied, demonstrating no difference in efficacy between RFA and recurrent surgical resection [58]. In a study by Chen et al., the 1- and 3-year OS for RFA was shown to be 95.8% and 71.4%, respectively, with a 1- and 3-year DFS of 85.9% and 64.1%, respectively [35]. The mRECIST complete response (CR) rate was 47%, with a partial response (PR) rate of 39% [36]. For NAFLD-HCC, RFA was shown to have similar overall outcomes in NAFLD-HCC patients specifically compared with HCC from other etiologies [37]. In contrast, Chin et al. demonstrated improved outcomes for curative treatments of NAFLD-HCC, including resection and OLT, compared with HCC from other etiologies [26].

Due to the high rates of comorbid conditions like diabetes and obesity in patients with NAFLD, the management of HCC in this patient population can be a challenge. This is because diabetic patients have been shown to have lower survival rates for patients undergoing ablation than those without DM2. Notably, metformin users among diabetic patients with HCC undergoing ablation has been shown to have a favorable OS compared with patients without treatment [59,60]. Additionally, with NAFLD-HCC presenting at a later stage than other etiologies of HCC, selection for ablative techniques is often limited, as ablation is most effective at early stages. Despite the technical challenge of ultrasonographic procedures in obese patients due to attenuation from subcutaneous and intrahepatic fat, a prospective observational study by Ohki et al. did not demonstrate a difference in outcomes after ablation between obese and non-obese patients [61]. The current advances with this treatment include studies investigating combination therapy with radiation therapy, immunotherapies, chemotherapies, and TACE [62].

**Table 3:** Advantages and disadvantages of LRTs [30].

Modality	Mechanism	Advantages	Disadvantages
Percutaneous ablation	Radiofrequency current, microwaves, or cycles of freezing and thawing which cause cell death.	Able to function as monotherapy for early-stage disease; fewer complications compared with transarterial therapies; potentially curative.	PAS, bleeding, iatrogenic injury, and cryoshock (cryoablation) [2,57].
TAE	Micro-embolic particles causing tumor ischemia.	Avoids ionizing radiation or systemic chemotherapy exposure; inexpensive.	PES, liver failure, abscess formation, and biloma [39].
TACE	Micro-embolic particles infused with chemotherapy causing a combination of tissue ischemia and chemotoxicity.	Higher radiologic response than TAE; well studied; first-line treatment for intermediate-stage HCC.	PES, liver failure, abscess formation, biloma, and systemic chemotherapy exposure [3].
TARE	Yttrium-90 beta-degradation causing focal cell death with ionizing radiation.	May be used early in disease with curative intent; higher quality of life compared with other transarterial therapies.	Radiation pneumonitis, fibrotic lung disease, RILD, liver failure, and abscess formation [63].

### 2.2. Microwave Ablation

#### 2.2.1. Overview

The most common thermal ablation technique is MWA, which uses electromagnetic energy in the 300 MHz to 300 GHz range to cause water dipoles to continually re-align with the applied field which causes heat production [64]. This results in this approach having better penetration into tissues, which allows for a larger zone of necrosis, the treatment of multiple tumor sites spontaneously, a reduced treatment time, and no heat sink effect that limits RFA [33].

#### 2.2.2. Indications and Complications

The official BCLC guidelines for NAFLD give the same recommendation for RFA and MWA [65], being recommended for HCC < 2 cm as standard therapy for non-transplant candidates without vascular or extrahepatic dissemination [20]. The characteristics of MWA offer unique theoretical benefits over RFA, which has resulted in it becoming the standard approach in most institutions. Another benefit of MWA is that, unlike RFA, there is no contraindication from having an implanted pacemaker or surgical clips.

The contraindications are similar to those of RFA: having a surgically resectable NAFLD-HCC and/or transplantation status, vascular invasion of the tumor, exophytic tumor location, uncorrectable coagulopathy, having a surgically resectable tumor and/or transplantation status, and intrahepatic biliary tree dilation. Unfortunately, the better penetration of the microwaves increases risk of damage to surrounding structures [66]. Adverse events for all percutaneous ablation procedures include bleeding, iatrogenic injury to local structures, post-embolization syndrome, and abscess formation [67].

#### 2.2.3. Outcomes

Despite these differences, several randomized trials have demonstrated similar outcomes between MWA and RFA [1,68]. In a study by Radosevic et al., it was demonstrated that while MWA resulted in a larger ablation zone, the overall efficacy was similar between the two [68]. As above, the future directions include combination therapies with intra-arterial approaches, including TACE and TARE [16], as well as with immunotherapies [62,69]. Violi et al. had a similar study, demonstrating a long-term progression (LTP) at 6% and 12% for WMA and RFA, respectively. This was not shown to be a statistically significant difference, however [70].

### 2.3. Cryoablation

#### 2.3.1. Overview

In CA, a needle is inserted into tissues, and liquid argon is passed through the cryoprobe, rapidly removing thermal energy from the surrounding tissues. The subsequent infusion of helium gas rapidly warms the probe, causing the thawing of the tissues. These cycles are then repeated. The mechanism of action is multifocal. CA causes direct cell death immediately through the freezing of the water within the tissues, causing cell shrinkage and dehydration. It then causes thawing through cell swelling and bursting. This is repeated multiple times to amplify this effect. Then, in a delayed phase, there is cytokine release, which causes apoptosis and ischemia [71].

#### 2.3.2. Indications and Complications

CA is another potentially curative treatment indicated for nonsurgical patients with small tumors, being demonstrated to be an effective treatment for HCC and metastatic colon cancer. The contraindications are similar to those of other ablative techniques. One limitation of CA is the risk profile, which includes parenchymal fracture, myocardial infarction, biliary fistula, hemorrhage, DIC, and ARDS, with a notable mortality rate of 1.5%, similar to the perioperative risk of hepatic resection. A unique complication is cryoshock, which is secondary to the release of cytokine release, which can cause renal failure secondary to myoglobinuria and coagulopathy [72].

#### 2.3.3. Outcomes

The measurement of outcomes is heterogenous, with some studies demonstrating improved outcomes of RFA over CA; recent studies demonstrate similar [66] or even improved outcomes with RFA, with Wang et al. showing CA resulting in a 1-, 3-, and 5-year LTP of 97%, 67%, and 40%, compared with the LTP in RFA of 97%, 66%, and 38%, respectively [73]. Glazer et al. demonstrated that CA was highly effective for liver tumors; however, this was significantly limited in efficacy below 4 cm [74].

## 3. Transarterial Embolization

### 3.1. Overview

**Figure 2:**
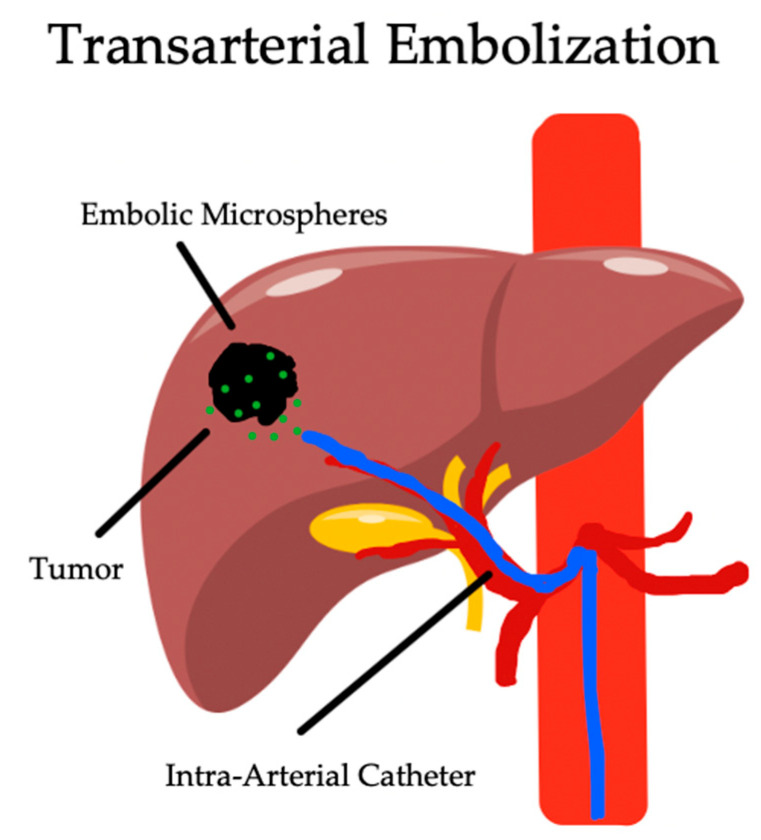
Image-guided embolization of hepatic arteries supplying tumor using embolic microspheres.

The efficacy of transarterial therapies for HCC relies on the dual blood supply of the liver, with the tumor primarily being supplied by the hepatic arteries. As seen on Figure 2, the selective isolation of the hepatic arteries feeding the tumor will preferentially damage the HCC over the surrounding parenchyma [75,76]. The efficacy of TAE relies on the physical embolization of these arteries, which directly induces hypoxia and cell death in the tumor cells. This is achieved by the selective catheterization of the hepatic artery feeding the tumor with the infusion of gel foam, polyvinyl alcohol, or microparticles ranging in size from 40 to 120 um [77]. This approach requires the catheterization of either a lobar hepatic artery for multifocal disease or a selective segmental artery for unifocal disease [78]. Unlike other intra-arterial therapies, this approach does not carry with it additional ways of inducing tumor necrosis, unlike chemotherapy for TACE and radiation for TARE. A direct comparison of the LRTs is displayed on Table 3.

### 3.2. Indications and Complications

TAE is indicated for nonsurgical patients who do not qualify for curative treatments. While the most benefit lies in BCLC stage B patients for a reduction in tumor burden and extending life expectancy, TAE is useful in BCLC stage C, with a higher benefit to BCLC stage B [38,79]. Additionally, it is effective in NAFLD-HCC BCLC A patients for maintaining eligibility on the transplant list through the Milan and UCLA criteria, with one study demonstrating a 78% success rate at 1 year [80]. The contraindications to TAE include decompensated cirrhosis (with a CPS of 8 or higher), reduced portal vein flow/portal vein thrombosis (PVT), a creatinine clearance of less than 30 mL/min, a high tumor burden, severe comorbidities, untreated esophageal varices, and elevated liver function markers [81]. In addition, for transarterial therapies, patients are required to be able to lay flat for prolonged periods of time, which is limited in patients with severe pain or heart disease [2].

While the complication rate in the immediate post-procedure period is lower than that for other transarterial therapies, the adverse events from TAE include off-target embolization/pulmonary shunting, abscess formation, biloma, and worsening liver failure [39]. As with all transarterial therapies, there is the risk of access site vascular injuries, of which a high BMI is a significant risk factor [82]. One complication common to transarterial therapies is post-embolization syndrome (PES), which results in abdominal pain, fever, and elevated liver function tests in the first 24–48 h post-embolization and spontaneously resolving in 7 to 10 days. The symptoms are secondary to ischemia, causing tumor necrosis with cytokine release. The risk factors include a tumor > 5 cm, multiple tumors, and variant patient anatomy. The treatment is supportive; however, several methods have been used to reduce the risk and severity of the symptoms. These include the administration of steroids, 5-HT3R antagonists, morphine, and midazolam and the intra-arterial administration of lidocaine [39,83]. Due to the risk of post-embolization abscess formation, perioperative antibiotics are frequently given to patients with a history of previous instrumentation or sphincter of Oddi dysfunction [84].

### 3.3. Outcomes

While research into the long-term outcomes of TAE is more limited than for TACE, there is strong evidence that TAE confers a significant survival benefit over conservative therapy in BCLC B patients [79,85]. Comparison with other transarterial procedures is more challenging. TACE was demonstrated to have a higher radiological response rate but the same PFS and OS as TAE [86]. Meyer et al. demonstrated a shorter time to progression (TTP) and higher rates of tumor recurrence and local response, with an overall lower mRECIST treatment response in TAE (CR was 18.4%; PR was 28.8%) compared with TACE (CR was 30.2%, and PR was 37.2%). The 1- and 2-year OS for TAE was 84.8 and 38.3% [82], and the PFS was 7.2 months [41]. Several studies showed similar results, with a longer TTP and higher rate of complete response in DEB-TACE over TAE [87,88]. However, others have failed to demonstrate a significant difference in the OS between the two for OS, PFS, and rates of downgrading BCLC B to gain OLT eligibility [88,89,90]. While the long-term survival for TAE may be inferior to TACE, the advantage of TAE in NAFLD-HCC lies in the immediate safety profile. Tsochatzis et al. demonstrated improved tolerability in HCC patients with borderline liver function [91]. Similarly, Agrawal et al. demonstrated a lower risk of PES in TAE compared to TACE, at 68.7% compared with 74.7%. They also found a significantly lower overall hospitalization stay, at 1.12 days compared with 1.47 days [39]. As a result, while TACE carries a 1A recommendation for NAFLD-HCC BCLC B [76], TAE is used frequently in select patients due to its improved toxicity profile [79,92].

## 4. Transarterial Chemoembolization

### 4.1. Overview

**Figure 3:**
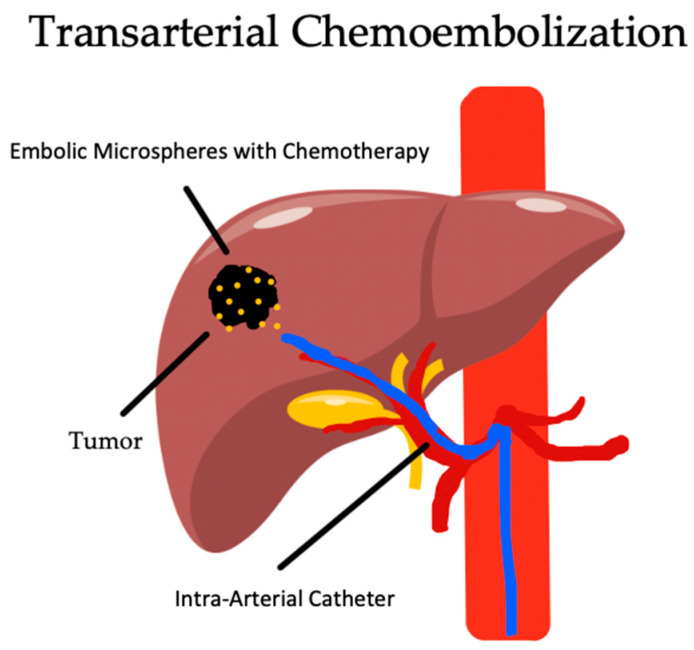
Image-guided transarterial chemoembolization of hepatic arteries supplying tumor utilizing chemotherapy-drug-eluting microspheres.

TACE utilizes the same localized ischemia effect as TAE, with the further benefit of the addition of a highly concentrated dose of chemotherapeutic drugs administered with the embolic agent. This is typically doxorubicin but is sometimes cisplatin or mitomycin C. This approach allows for the administration of a higher local dose of chemotherapy than what would be tolerated if given systemically [93]. A diagram of TACE can be seen below in Figure 3. This also results in a synergistic effect with the two mechanisms against NAFLD-HCC, with the localized ischemia inducing the production of VEG-F, which increases vessel permeability which increases the delivery of the chemotherapeutic [94]. Additionally, due to blood stasis, this reduces the local drug clearance, which improves the systemic safety profile [3,95].

Within TACE, there are two major approaches, conventional TACE (c-TACE) and drug-eluting bead TACE (DEB-TACE). In both cases, the first step of the procedure is the same as TAE, with the selective catheterization of either the lobar hepatic arteries for multifocal disease or the segmental hepatic artery feeding a solitary nodule [78]. In c-TACE, chemotherapy is emulsified into lipiodol oil, which has the benefit of being inexpensive, easily accessible, and well studied [67]. A downside of this approach is the washout of the chemotherapeutic agent, with a recent study showing that post-embolization plasma drug levels used in c-TACE soon approximated similar levels in systemic chemotherapy [96]. In contrast, the DEB-TACE comprises polyvinylchloride microspheres infused with the chemotherapeutic agent, which then slowly release the drug, which has the theoretical benefit of reducing systemic chemotherapy exposure [2].

### 4.2. Indications and Complications

While TACE may be considered for BCLC A patients to maintain transplant status or downgrade as a bridge to resection or transplant [97], it is considered a grade 1A recommendation in the treatment for intermediate, unresectable NAFLD-HCC BCLC stage B patients [42]. Additionally, it may be used for unilateral portal vein embolization, to induce contralateral liver lobe hypertrophy before surgical resection [98]. TACE is also used in BCLC stage C patients for palliation and local tumor control, frequently in concert with systemic chemotherapy, which is an area of active research [67]. Absolute contraindications include decompensated cirrhosis (CPS 8 or higher), reduced portal vein flow, creatinine clearance < 30 mL/min, bi-lobar tumor involvement, and technical infeasibility. The relative contraindications include a high tumor burden, severe comorbidities, untreated esophageal varices, and elevated LFTs [2]. Notably, lobar and selective/segmental TACE is frequently performed if the bilirubin is up to 3 or 4 mg/dL; however, the AASLD recommends against TACE if the bilirubin is greater than 3 mg/dL, there is PVT, or unless segmental treatment is possible [99]. However, one study did demonstrate a significant survival improvement with TACE in patients with either segmental branch or first-order branch PVT [100].

The complications are similar to those of TAE and include the deterioration of liver function, including the development of ascites or liver failure. Iatrogenic injury is also possible, including the development of bile duct injury, biloma, ischemic cholecystitis, iatrogenic dissection, or access site hematoma. Liver abscess may also occur, especially in patients with recent biliary instrumentation or sphincter of Oddi dysfunction [77]. Of note, while obesity is a well-known risk factor for postoperative complications, an elevated BMI was associated with an improved OS in TACE. This, however, is likely secondary to the end-stage cachectic effects of HCC. Similar to TAE, post-embolization syndrome is common, with rates ranging from 6.2% to >80% [99]. Overall, the combined risk of major complications occurs in 5% of patients, with a risk of death of ~1% [101].

### 4.3. Outcomes

In comparison with conservative measures, both c-TACE and DEB-TACE have consistently shown strong evidence for improved outcomes in patients appropriately selected [95,102,103]. According to Burrel et al., the 1-, 3-, and 5-year survival for DEB-TACE was 89.9%, 66.3%, and 38.3%, respectively, with a median survival time of 48.6 months in recurrent BCLC A and BCLC B [43]. In a study by Kudo et al., the PFS was 13.5 months [44]. In a study by Meyer et al., the mRECIST CR was 47.3%, and the PR was 67.4% [41]. In patients with NAFLD-HCC, a large retrospective review by Young et al. demonstrated similar treatment efficacy compared with other etiologies of HCC [45]. However, in a retrospective study by Wu et al., obesity was associated with higher residual disease, new lesions, and progressive disease in patients with HCC treated with TACE [104]. Concerning the choice of DEB-TACE compared with c-TACE, the evidence is mixed. In the PRECISION V study, there was an increase in tumor response, a reduction in severe hepatotoxicity, 6-month disease control, and lower doxorubicin-related adverse events in the DEB-TACE group relative to the c-TACE group [95]. Despite this, multiple studies have demonstrated a similar OS between the two approaches [105,106]. The safety profile between these options is also in question, despite the reduced systemic exposure of chemotherapeutics in DEB-TACE [95].

## 5. Transarterial Radioembolization

### 5.1. Overview

**Figure 4:**
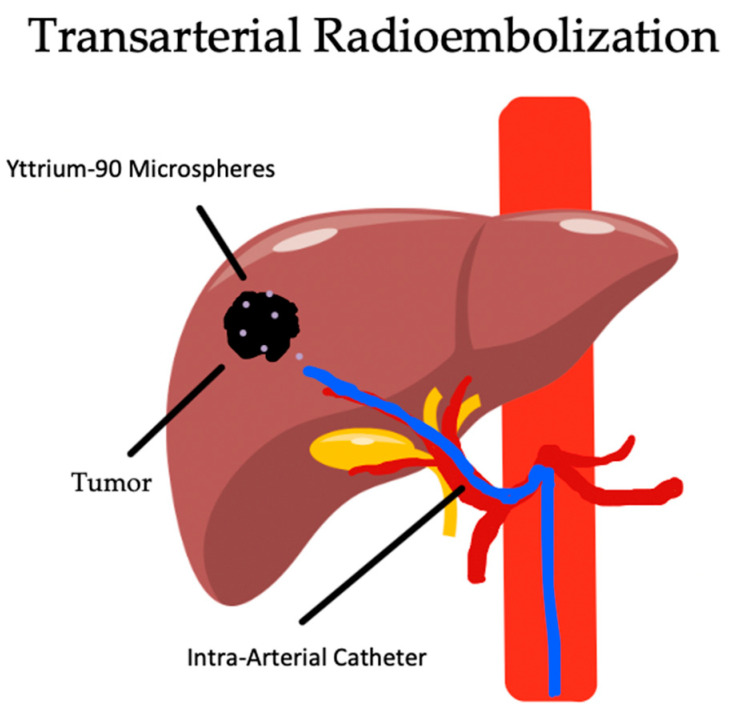
Image-guided transarterial radioembolization of hepatic arteries supplying tumor with Y-90 microspheres.

In TARE, also known as Selective Internal Radiotherapy (SIRT), instead of chemical or ischemic injury, the primary mechanism of tumor lysis is through the release of beta particles from the degradation of yittrium-90 (Y-90) into zirconium-90. Radioactive Y-90 is bound to an embolic agent, either resin or glass, which is selectively embolized into the tumor through the supplying hepatic arteries. This is illustrated on Figure 4 below. Unfortunately, if these particles bypass the tumor capillary bed and are released into the caval system, the patient is at high risk of radiation pneumonitis and fibrotic lung disease. Because of this, a separate procedure is performed 1–2 weeks before the TARE procedure, during which the hepatic arteries are mapped, with quantification of the hepatopulmonary shunt fracture. This is performed with angiography, where microaggregate albumin radiolabeled with Technetium-99m is selectively released in the artery feeding the HCC, which is then promptly imaged by SPECT imaging [107]. The dosing is then calculated, depending on the type of microspheres used, tumor load, lung shunt fraction, and three-dimensional tumor volume [79,108]. After the subsequent therapeutic procedure, the patient is re-imaged after 3–6 months to monitor for response, which is more delayed follow-up imaging than TAE and TACE [109].

### 5.2. Indications and Complications

The first-line treatment for NAFLD-HCC BCLC stage B remains TACE; however, recommendations from the American Association for the Study of Liver Diseases (AASLD) and National Comprehensive Cancer Network (NCCN) do not state that TARE is inferior to TACE in the treatment for unresectable BCLC stage B [46]. Otherwise, the indications for TARE remain similar to those for TACE, including downsizing HCC deemed borderline in resection [47,110] and neoadjuvant lobectomy to preoperatively increase the liver function of the contralateral liver parenchyma [2]. One unique advantage of TARE is the nearly negligible ischemic burden due to much fewer embolic particles, which allows it to be used in BCLC C patients with PVT [111], unlike in TAE and TACE. The most notable contraindication to TARE is determined during the preceding mapping procedure as a lung shunt fraction of >20% or significant hepatoenteric shunting [63]. Other contraindications include bilirubin greater than 2 mg/dL, encephalopathy, and prior radiation to the liver [109].

One of the benefits of TARE is the improved safety profile, again secondary to the reduced immediate ischemic effect of large-scale capillary embolization. While the most common clinical toxicities include fatigue (57%), pain (23%), and nausea/vomiting (20%) [48], TARE has been shown to have an improved toxicity profile [112], with studies demonstrating an overall improved quality of life post-treatment relative to TACE [113]. One unique late complication of TARE is radiation-induced liver disease (RILD), which consists of local vascular, fibrotic, and parenchymal change secondary to radiation exposure. It presents with jaundice, ascites, and increasing liver function tests 4–8 weeks after treatment and is associated with a high mortality rate [114].

### 5.3. Outcomes

The outcomes for TARE in appropriately selected patients are excellent. According to a large retrospective study by Salem et al., the OS from TARE in Child–Pugh A patients was 47.3 months and 27 months in Child–Pugh B patients [115]. In a meta-analysis by Rognoni et al., the 1- and 3-year OS was 63% and 27%, respectively [49]. The mRECIST CR and PR were 13.7% and 43.1%, respectively [51,116]. TARE appears to be equally effective for the treatment of NAFLD-HCC when compared with other causes of HCC, as measured by OS and PFS [52]. Similar outcomes were also shown by Schotten et al. in a comparison of TARE for the treatment of NAFLD-HCC vs. HCC from HBV. This demonstrated an OS of 11.1 months and 9.3 months, respectively, which was not found to be significantly different (*p* = 0.38) [53].

Concerning comparison with TACE, the prospective PREMIERE trial demonstrated TARE to have a TTP of 14.5 months, compared with 6.4 months, *p* = 0.0019. This trial did, however, demonstrate no significant difference in the OS (23.8 months vs. 17.7 months, *p* = 0.9772) compared with TACE for intermediate BCLC patients [50]. A meta-analysis by Lobo et al. also demonstrated similar survival with TACE [117]. In comparison with bridging to transplant, TARE appears to outperform TACE, with an RCT by Lewandowski et al. demonstrating an improved TTP, at 18.2 months for TACE and 33.3 months for TARE. Additionally, TARE has also been shown to have higher quality of life post-embolization, including having the added benefit of requiring two procedures instead of the typically recurrent TACE procedures [113,118].

## 6. Future Directions

There are several key developments actively in progress which are promising for the future of LRTs for NAFLD-HCC. The overlap of LRTs with immune system modulators is one area of research that has potential, as one of the effects of LRTs is the immune response that is elicited through the localized cell death produced [116]. There is thus a push for the incorporation of immune checkpoint inhibitors earlier in the disease process [119]. In one retrospective study by Raj et al., they assessed the combination of any LRT, including TACE, TARE, and ablation, with neoadjuvant atezolizumab/bevacizumab, demonstrating 11% with a complete response, with 1-, 3-, and 5-year survival rates of 100%, 91%, and 81.8%, respectively [116]. In a study by Duffy et al., there were promising initial results with the addition of tremelimumab in combination with ablation for patients with advanced HCC [120]. One large prospective trial investigating the combination of LRTs and immunomodulators is the IMbrave050 trial. This phase III trial is investigating the addition of atezolizumab plus bevacizumab to resected or ablated high-risk HCC by Qin et al., with preliminary data showing a promising improvement in PFS. Another promising trial is EMERALD-1, which has shown significantly improved PFS with the addition of durvalumab and/or bevacizumab with TACE in patients with unresectable HCC who qualify for embolization [121]. While the efficacy of immune therapy for HCC in patients with NASH may be limited [28], it is unknown whether the combination of LRTs and immunotherapy has different outcomes in NAFLD-HCC relative to other etiologies of HCC. This question is an opportunity for future work.

One field demonstrating remarkable progress is in the field of NAFLD-HCC prevention. Recently, the FDA approved the use of the thyroid-hormone receptor-beta agonist resmetirom to reduce the risk of the development of cirrhosis in patients with NAFLD, with a demonstrated resolution of NASH in 30% of those receiving the high dose relative to the 10% in the placebo group in phase III trials [122,123]. Another promising treatment entering phase III trials is Lanifibranor, a Pan-PPAR agonist which demonstrated a resolution of NASH without the worsening of fibrosis in 49% of those receiving the high dose relative to 22% in the placebo group [124,125]. Other novel approaches, still in pre-clinical testing, include loading drugs besides conventional chemotherapeutic drugs onto drug-eluting beads (DEBs) for TACE, including tyrosine kinase inhibitors, bevacizumab, and a combination of doxorubicin and sigma-2 receptor agonist SW43 [126,127].

Another promising development is histotripsy, the first noninvasive, nonionizing, and nonthermal ablative locoregional therapy. In this procedure, a specialized probe on the skin surface creates microsecond bursts of targeted ultrasound waves. This destroys tissue by repeatedly generating and collapsing microbubbles within the tumor, resulting in the mechanical reduction of the tumor to acellular debris. The multicenter phase I THERESA Study demonstrated preliminary evidence of reassuring safety and efficacy in tumor destruction [30,128]. In addition to the demonstrable reduction in treated tumor size, early results showed efficacy in non-treated tumors, indicating an immune abscopal effect due to exposure of tumor antigens. The prospective, multicenter, single-arm HOPE4LIVER Trial is the next step, with early results by Mendiratta-Lala et al. demonstrating the technical success of 42 of the 44 treated tumors and procedure-related major complications occurring in three of the forty-four participants, both meeting the performance goal. The FDA has recently approved this technology for early market use, with further work needed to elucidate the clinical outcomes of this approach [129].

## 7. Conclusions

As the prevalence of obesity continues to grow, NAFLD is expected to become the most common cause of HCC in the US by 2030 [9]. While liver transplantation is the most effective treatment, very few NAFLD-HCC patients qualify for this due to their comorbidities or advanced stage at diagnosis. This has resulted in a 5-year survival rate of less than 20% [95]. For nonsurgical candidates, which make up most cases, LRTs serve as the mainstay of treatment. Percutaneous ablation stands as the only curative intent treatment and has similar outcomes to surgical resection in early-stage disease. TACE is the first-line therapy for intermediate-stage nonresectable HCC, with TAE having similar outcomes with a more tolerable short-term toxicity profile. TARE has similar outcomes, an improved side effect profile, and improved outcomes for PVT and downstaging patients to qualify for transplant. It has not been demonstrated that the outcomes for LRTs differ significantly in NAFLD-HCC relative to other etiologies. Finally, there are new promising treatments in development, including combining immunotherapy with LRTs, NAFLD-HCC prevention, and histotripsy.

## Figures and Tables

**Table 1:** Barcelona Clinic Liver Cancer staging and indicated treatment for HCC [2,20].

Stage	BCLC 0	BCLC A	BCLC B	BCLC C	BCLC D
Severity	Very early stage	Early stage	Intermediate stage	Advanced stage	Terminal stage
Definition	Single < 2 cm, Child–Pugh A/B	Less than 3 nodules of <3 cm, Child–Pugh A/B	Multinodular, Child–Pugh A/B	Portal invasion and/or extrahepatic spread, Child–Pugh A/B	Any tumor burden if Child–Pugh C
Treatment	Resection; if nonsurgical candidate, ablation	Resection/OLT; if nonsurgical candidate, ablation	TACE/TARE/TAE	Systemic therapy; Possible TACE/TARE/TAE	Supportive care
